# Rapamycin Prolongs the Survival of Corneal Epithelial Cells in Culture

**DOI:** 10.1038/srep40308

**Published:** 2017-01-05

**Authors:** Sanaz Gidfar, Farnoud Y. Milani, Behrad Y. Milani, Xiang Shen, Medi Eslani, Ilham Putra, Michael J. Huvard, Hossein Sagha, Ali R. Djalilian

**Affiliations:** 1 Department of Ophthalmology and Visual Sciences, University of Illinois at Chicago, Chicago IL, USA.

## Abstract

Rapamycin has previously been shown to have anti-aging effects in cells and organisms. These studies were undertaken to investigate the effects of rapamycin on primary human corneal epithelial cells *in vitro*. Cell growth and viability were evaluated by bright field microscopy. Cell proliferation and cycle were evaluated by flow cytometry. The expression of differentiation markers was evaluated by quantitative PCR and Western blot. Senescence was evaluated by senescence-associated β-Galactosidase staining and by Western blot analysis of p16. Apoptosis was evaluated by a TUNEL assay. The results demonstrated that primary HCEC treated with rapamycin had lower proliferation but considerably longer survival *in vitro*. Rapamycin-treated cells maintained a higher capacity to proliferate after removal of rapamycin and expressed more keratin 14, N-Cadherin, DeltaNp63 and ABCG2, and less keratin 12, consistent with their less differentiated state. Rapamycin treated cells demonstrated less senescence by X-β-Gal SA staining and by lower expression of p16. Apoptosis was also lower in the rapamycin treated cells. These results indicate that rapamycin treatment of HCEC prevents the loss of corneal epithelial stem/progenitor cells to replicative senescence and apoptosis. Rapamycin may be a useful additive for *ex vivo* expansion of corneal epithelial cells.

The corneal epithelium is the outermost layer of the cornea. Epithelial stem cells located primarily at the corneal limbus continually renew the corneal epithelium by first differentiating into transient amplifying cells (aka progenitor cells) and then migrating centripetally and anteriorly to undergo terminal differentiation into mature epithelial cells^1^. The integrity of the corneal epithelium depends on the continual function of these self-renewing stem cells. However, they are sensitive to physical or chemical insults, which may compromise their capacity to divide and repopulate the corneal epithelial surface^2^,^3^,^4^. Clinically, this can manifest in a condition known as limbal stem cell deficiency where the corneal epithelium is partially or totally replaced by conjunctival epithelium^5^.

In recent years, there has been increasing interest in growing corneal epithelial cells in culture for research and therapeutic applications^6^,^7^,^8^. A common problem with culturing cells is preserving their proliferative potential over time. Therefore, many investigators have used feeder cells such as growth arrested 3T3 fibroblasts to help maintain and induce proliferation of limbal stem/progenitor cells in culture. Although these methods have successfully addressed some of the inherit limitations of culturing cells, improvements of the culture process are desired^9^.

The mammalian Target of Rapamycin (mTOR) is a serine/threonine protein kinase that regulates many cellular functions such as growth, proliferation, metabolism, and aging^10^. Increased or persistent activity of mTOR accelerates epithelial cell senescence and causes them to exit the proliferative cell pool^2^,^11^,^12^. Increased mTOR activity has also been linked with epidermal stem cell exhaustion and premature ageing^12^. Rapamycin, also known as sirolimus, is a commonly used immunosuppressant whose primary mechanism of action is through inhibition of mTOR. Rapamcyin has been shown to extend lifespan in yeast, nematodes, fruit flies, and mice^13^. Rapamycin-inhibition of mTOR can protect oral mucosal epithelial progenitor cells from replicative senescence and extend their lifespan *in vitro*^2^.

In the cornea, mTOR plays an important role in regulating scarring, neovascularization, and inflammation^14^,^15^,^16^,^17^. Inhibiting mTOR with rapamycin can decrease corneal scarring in part by inhibiting the proliferation and differentiation of corneal myofibroblasts^18^. The specific effects of rapamycin on corneal epithelial cells, however, are not known. In this study, we investigate how rapamycin affects corneal epithelial cells *in vitro*. We have examined whether rapamycin can prolong the survival of corneal epithelial cells *in vitro* and have investigated its specific effects on proliferation, differentiation and replicative senescence.

## Results

### Rapamycin prolongs corneal epithelial cell survival *in vitro*

**Figure 1:**
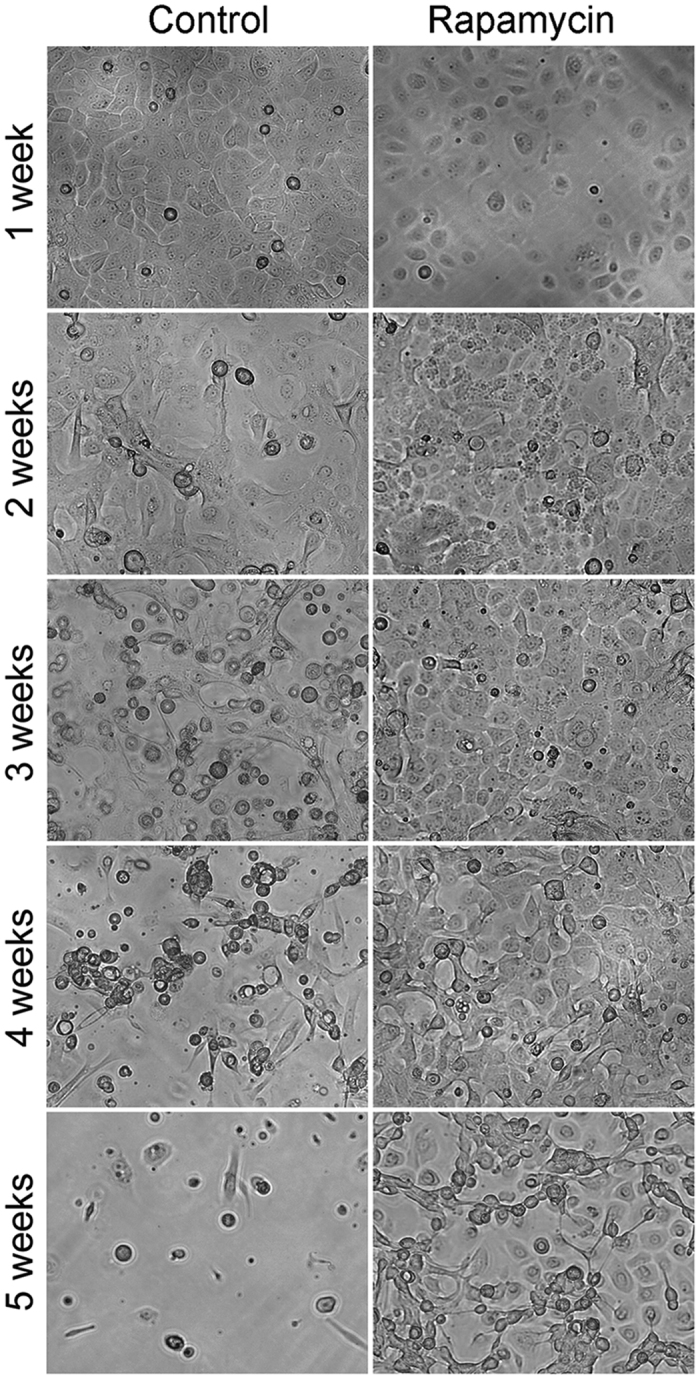
Bright field microscopy of primary human corneal epithelial cell cultures treated with or without rapamycin. Rapamycin significantly prolongs the survival of cells *in vitro*. Scale bar, 50 μm.

Primary HCEC were grown in serum free conditions while being exposed continuously to rapamycin or the vehicle (DMSO). Bright field microscopy revealed that rapamycin treated cells persisted up to five weeks while control cells began to show signs of differentiation by week three with very few cells visible by week five (Fig. 1).

### Rapamycin decreases proliferation and alters the cell cycle in primary HCEC

**Figure 2:**
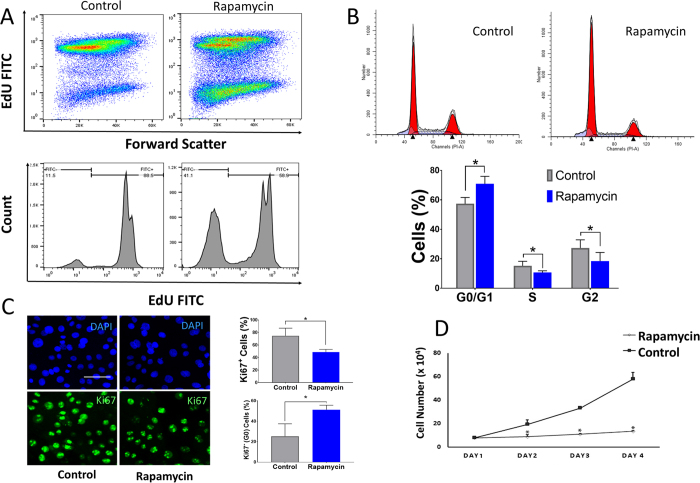
Rapamycin decreases proliferation and alters the cell cycle. Distribution of EdU positive cells after treatment with rapamycin versus control (58.90% vs. 88.50%; *P* < 0.001) (**A**). Rapamycin increased the percentage of cells in G0/G1 phase compared to control and decreased the percentage of cells in S and G2 phases compared to control (**B**). Immunostaining for the proliferation marker Ki67 in cells treated with rapamycin versus control. The percentage of cells in the G0 phase was calculated indirectly based on the fact that Ki-67 is present during in all the active phases of the cell cycle (G1, S, G2, and mitosis), but is absent from resting cells (G0). (**C**) Effect of rapamycin on the proliferation of HCEC. (D) Scale bar, 20 μm. Error bars represent mean ± standard deviation among experiments. (*P < 0.01).

The rate of proliferation in primary HCEC was measured using an EdU assay (analogous to a BrdU incorporation assay). Cells treated with rapamycin demonstrated lower EdU incorporation, hence lower proliferation rate, compared to control cells (58.90% vs. vehicle 88.50%; *P* < 0.001 (Fig. 2A). The change in proliferation with rapamycin paralleled the results of the cell cycle analysis. In particular, rapamycin treated cells demonstrated a decrease in the percentage of cells in the G2 phase (18.43 ± 5.85% vs. vehicle 27.35 ± 5.52%) while the percentage of cells in G0/G1 phase was higher (70.90 ± 5.11% vs. vehicle 57.36 ± 4.27%). The percentage of cells in S phase for rapamycin treated cells were 10.66 ± 1.22% vs. vehicle 15.27 ± 3.04% (Fig. 2B) (all comparisons; *P* < 0.01). Ki67 was also used for measuring proliferation. Results showed that proliferation was decreased in rapamycin treated cells compared to control (74.75% vs. 48.75%) (*P* = 0.028). There were higher cells in G0 cell cycle in rapamycin treated cells compared to the control (25.25% vs. 51.25%) (*P* = 0.007) (Fig. 2C). To determine whether rapamycin decreases the proliferation of HCEC, we serially counted the number of cells in parallel plates exposed to rapamycin or DMSO. Our results demonstrate that rapamycin markedly lowered the rate at which cell numbers increased in culture (Fig. 2D) (*P* < 0.001).

### Rapamycin treated HCEC have higher capacity to proliferate and express more limbal markers

**Figure 3:**
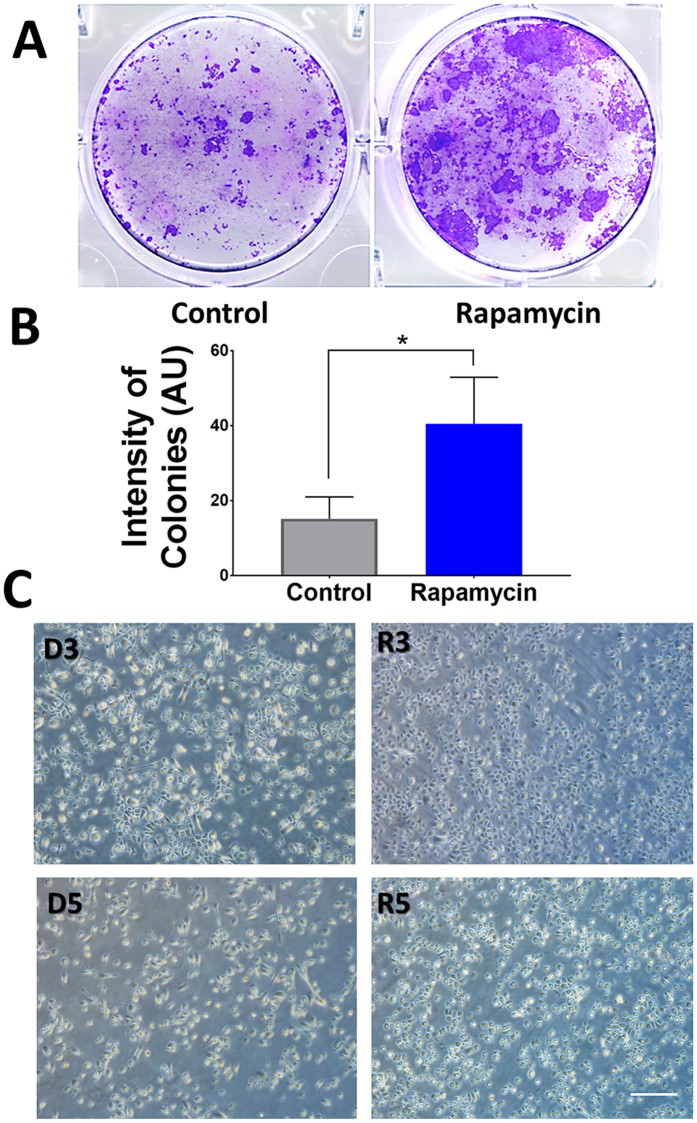
Rapamycin significantly increases the capacity for proliferation. Cells were pretreated with or without rapamycin, and then their capacity for proliferation was measured after removal of rapamycin (**A**). Treatment with rapamycin significantly increased the capacity for proliferation after removal (**B**). HCEC in rapamycin could be passaged up to three (R3) and five times (R5) compared to DMSO where they became more spindle starting in passage three (D3) and further in passage five (D5) (**C**). Scale bar, 100 μm. Error bars represent mean ± standard deviation among experiments. (*P = 0.007).

Terminal differentiation is another mechanism that limits the lifespan of epithelial cells in culture^19^. With increasing time in culture, epithelial cells typically begin to differentiate and stack on top of each other. To functionally assess the presence of limbal stem/progenitor cells we measured their capacity to proliferate after rapamycin treatment. Primary HCEC were treated with rapamycin or control for 10 days after which the cells were transferred onto new plates and allowed to form colonies (without additional exposure to rapamycin). As shown in Fig. 3A,B, cells previously treated with rapamycin demonstrated significantly higher capacity to proliferate after removal of rapamycin, both in terms of colony size and number (*P* = 0.0069). Similarly, rapamycin treated HCEC could be passaged up to five times compared to three times in control before the cells begin to lose their cuboidal shape and become more spindle-like (Fig. 3C).

**Figure 4:**
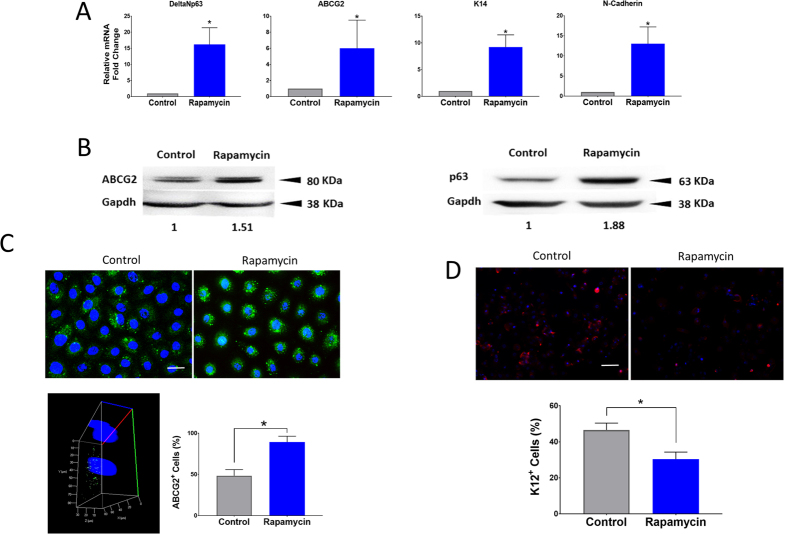
Rapamycin increases the expression of limbal markers associated with a less differentiated state. Quantitative RT-PCR of DeltaNp63, ABCG2, K14, N-Cadherin in human corneal epithelial cells treated with and without rapamycin showed a significant increase in the rapamycin treated group (**A**). Western blot analysis (cropped blot) showed an increase in the stem cell markers p63 and ABCG2 with rapamycin treatment (**B**). HCEC treated with rapamycin demonstrated a higher percentage of ABCG2 positive staining cells compared to control *(P* < 0.001). Confocal imaging further confirmed that the staining is limited to the apical surface of the cells (**C**). Immunostaining for the differentiation marker K12 in cells treated with rapamycin versus control. Rapamycin treatment resulted in less K12 + cells compared to control (**D**). Scale bar, 20 μm. Error bars represent mean ± standard deviation among experiments. (*P < 0.001).

The expression of limbal epithelial markers DeltaNp63, ABCG2, keratin (K)14 and N-Cadherin was higher in the rapamycin treated cells in comparison to control (Fig. 4A). Western blot also confirmed that expression of p63 and ABCG2 was higher after rapamycin treatment (Fig. 4B). HCEC treated with rapamycin demonstrated a higher percentage of positive staining for ABCG2 (*P* < 0.001) and lower percentage of positive staining cells for K12 compared to control cells (*P* < 0.0001) (Fig. 4C,D).

### Rapamycin decreases replicative senescence and apoptosis in HCEC

**Figure 5:**
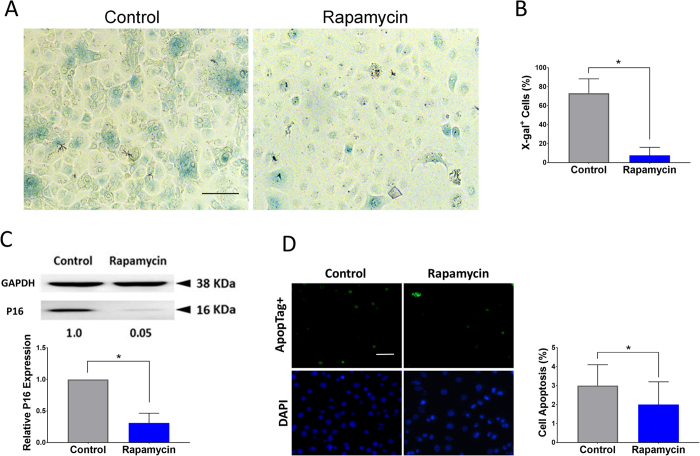
Rapamycin decreases senescence and apoptosis *in vitro*. Senescence-associated beta-galactosidase (X-β-Gal SA) staining of HCEC treated with and without rapamycin after three weeks (**A**). X-gal staining showed a significantly lower number of senescent cells in the group treated with rapamycin at week three. Scale bar, 100 μm (**B**). Western blot analysis (cropped blot) showed that rapamycin treated cells have lower expression of p16 (**C**). TUNEL assay of HCEC treated with or without rapamycin at three weeks. Less TUNEL positive cells were observed in rapamycin-treated group compare to control (**D**). Scale bar, 20 μm. Error bars represent mean ± standard deviation among experiments. (*P < 0.001).

Replicative senescence is one of the mechanisms that limits the lifespan of epithelial cells in culture^20^. Therefore, the effect of rapamycin on the senescence of corneal epithelial cells was evaluated using senescence-associated beta-galactosidase (X-β-Gal SA) staining^21^. As noted in Fig. 5A, cells treated with rapamycin demonstrated significantly lower number of senescent cells compared to the control group at three weeks (*P* < 0.000005) (Fig. 5B). Likewise, the expression of senescence marker p16 was lower in the rapamycin treated cells by Western blot (Fig. 5C). Since X-gal staining can also increase in apoptotic cells^22^, a TUNEL assay was used to evaluate apoptosis more specifically. The results indicted a lower percentage of apoptotic cells in rapamycin treated HCEC compared to control (Fig. 5D,E, *P* < 0.01).

### Rapamycin treated HCEC have reduced inflammatory response

**Figure 6:**
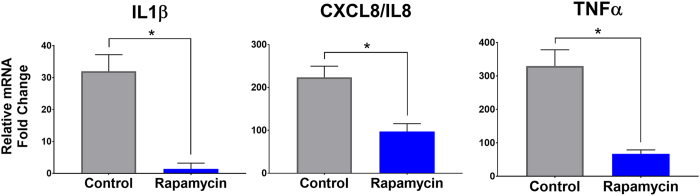
Rapamycin decreases the response to inflammatory stimulation. Quantitative PCR of IL1β, IL8, TNFα in human corneal epithelial cells after stimulation of TLR3 with Poly IC showed a significantly lower induction of inflammatory genes in the rapamycin treated group. Error bars represent mean ± standard deviation among experiments. (*P < 0.001).

Rapamycin has previously been shown to have anti-inflammatory properties^23^. The effect of Toll-like receptor (TLR)-3 activation in HCEC treated with or without rapamycin was evaluated. As shown in Fig. 6, the expression of tumor necrosis factor (TNF)-α, Interleukin (IL)-1β, and IL-8 was significantly lower in the rapamycin treated cells compare to control.

## Discussion

In this study we have demonstrated that rapamycin prolongs the survival of human corneal epithelial cells *in vitro.* Rapamycin treatment was found to keep the cells in a less differentiated and less proliferative state resulting in less replicative senescence and less apoptosis. The concept of senescence was formally described by Hayflick *et al*. as the finite replicative lifespan of human fibroblasts in culture^24^. Specifically, there is a limit to how many times a mitotic cell could divide before it loses its replicative potential, and this withdrawal from the cell cycle is called replicative senescence. Today, we know that there are many triggers of senescence, including telomerase dysfunction, DNA damage, disrupted chromatin, and strong mitogenic signals^25^. P53 and p16/Rb are thought to be the key mediators of senescence^26^. Senescence plays an essential anticancer role by halting the proliferation of cells with chromosomal aberrations that may undergo malignant transformation. Prior research has demonstrated that rapamycin-inhibition of the mTOR pathway delays senescence and the aging process, notably in other epithelial cells^2^,^14^.

Of note, cells in culture may be subject to contact inhibition (CI), a phenomenon of reversible cell cycle arrest due to a high cell density. A recent study shows that CI may be due to inhibition of the mTOR pathway and suppression of p21-induced senescence^27^. Due to the complexity of the mTOR pathway, however, there are likely multiple mechanisms responsible for its effect on senescence. Another closely related mechanism that may also contribute to the prolonged survival of the rapamycin treated cells, is the inhibition of apoptosis^28^.

More recent studies have found that rapamycin may also hold stem cells in a quiescent state^29^. They reported that mTORC1 activation is a necessary and sufficient criterion for the transition of stem cells from G_0_ into G_Alert_, a phase that represents an adaptation of cells under injury or stress to more quickly reenter the cell cycle. Inhibition of mTORC1 would conceivably force stem cells to remain in the fully quiescent G_0_ state, which is consistent with our observation that rapamycin-treated cells exhibited less proliferation and differentiation. Similarly, we found rapamycin treated cells had higher percentage of cells in the G0/G1 phase. These results support the idea that rapamycin may hold cells in a quiet state where cells are less differentiated and proliferate less.

Given our results, rapamycin may be a useful additive for *ex vivo* expansion of corneal epithelial cells. In particular, rapamycin may be used to prolong the survival and preserve the proliferative potential of corneal epithelial cells in culture. Likewise, rapamycin may have a possible therapeutic role in chronic epithelial disorders that involve accelerated cell loss or premature senescence. Dry eye, for example, is characterized by an increase in tear film osmolarity and chronic hyperosmotic stress in the corneal epithelial cells^30^. It is known that hyperosmotic stress causes cellular changes that may induce corneal epithelial inflammation and apoptosis^31^,^32^,^33^. Recent studies highlight the role of mTOR in corneal scarring, neovascularization, and inflammation^19^,^34^. An ocular formulation of rapamycin is being evaluated in clinic trials for posterior segment disease, raising promise for potential future anterior segment applications^35^,^36^,^37^.

One of the limitations of the methodologies used in this study is that due to differences in the rate of proliferation, the rapamycin and control cultures would always end up with different cell densities. The cell density itself can have an effect on the rate of apoptosis and senescence. However, there is no easy way to control for this indirect effect of cell density.

In conclusion, these results suggest that rapamycin, an inhibitor of mTOR, prolongs the survival of corneal epithelial cell *in vitro* and maintains their proliferative potential. This finding may prove useful for growing corneal epithelial cells in culture. Further studies are needed to determine the mechanisms by which mTOR regulates corneal epithelial stem/progenitor cells.

## Methods

### Human Corneal Epithelial Cell Culture

Primary human corneal epithelial cell (HCEC) cultures were initiated from more than 100 different cadaver corneas (age range 17–88) kindly provided by the Illinois (Chicago, IL, USA) and Midwest Eye Banks (Ann Arbor, MI, USA). The 1.5-mm limbal rings were treated with Dispase (2 mg/mL; Gibco, Grand Island, NY, USA) at 37 °C for two hours to separate the epithelial sheets, then digested in 0.25% trypsin-EDTA for five to ten minutes. Cells were washed and resuspended in keratinocyte serum-free medium (Invitrogen, Grand Island, NY, USA) and plated in collagen-coated tissue culture plates. Cells from passage zero were used for all of our experiments. HCEC were treated with 2 nM Rapamycin (Sigma-Aldrich, USA) or vehicle control [dimethyl sulfoxide] (DMSO maximum concentration 0.04%) beginning when the cells had reached a confluency of approximately 15–20% and was continued for up to five weeks. The media was changed every 1–2 days and the cells were serially examined and photographed under bright field microscopy (Leica DMi1) using LAS V4.5 software. Viability of the cells at various times was examined using Trypan blue staining.

### Western Blot Analysis

Cells cultured on 100-mm dishes were rinsed twice with PBS and harvested in SDS RIPA buffer (Sigma-Aldrich, USA) supplemented with protease/phosphatase inhibitors (Sigma-Aldrich, USA). After protein concentration measurement, equal amounts of each sample were mixed with 2× Laemmli buffer (Bio-Rad Laboratories) and 5% beta-mercaptoethanol (Sigma-Aldrich, USA), denatured by heating at 95 °C for 10 minutes, and subjected to electrophoresis on 4% to 20% Tris-Glycine gels (Invitrogen, Grand Island, NY). The protein bands were transferred to polyvinylidene difluoride membranes. The membranes were incubated in 5% BSA in Tris-buffered saline (TBS) for 1 hour followed by an overnight incubation (4 °C) with primary antibodies at the optimal concentration. The membranes were washed with TBS with 0.03% Tween 20 and incubated with the horseradish peroxidase (HRP)–conjugated secondary antibody for 1 hour at room temperature. Detection was performed with ECL Plus Western Blotting Detection System (Amersham, Buckinghamshire, UK). The following antibodies were used: mouse anti-human p16 (554079) (BD Pharmingen), p63 Ab-1(4A4) mouse MAb (NeoMarkers, Fermont, CA), ABCG2 rabbit mAb (42078) (Cell Signaling, Danvers, MA), GAPDH (D16H11) (Cell Signaling, Danvers, MA). Gels were run under the same experimental conditions to allow for direct comparison of bands.

### EdU Proliferation Assay

For the proliferation assay, the Click-iT EdU Alexa Fluor 488 Flow Cytometry Assay Kit (Thermofisher) was used to measure cell proliferation as described in the manufacturer’s manual. In brief, HCEC were treated with either 2 nM rapamycin or control [dimethyl sulfoxide] (DMSO maximum concentration 0.04%) starting at 10–15% confluency and continued for one week. To assess the proliferation, 20 μM 5-ethynyl-2′-deoxyuridine (EdU) was added to HCEC over night. Cells were harvested from tissue culture plates by incubating in 0.25% trypsin, 2.21 mM EDTA (Sigma-Aldrich). They were subsequently washed twice in PBS/1% BSA, fixed in 100 μl Click-iT fixative and washed twice in 1× saponin-based permeabilization and wash reagent. The Click-iT EdU reaction cocktail (1×) was prepared according to the manufacturer’s instructions and added to the cell pellet. Samples were incubated for 30 minutes at room temperature in the dark, and washed with 1× saponin-based permeabilization and wash reagent. They were analyzed using a fluorescent-activated cell sorting (BD LSR Fortessa) and quantified with FlowJo V10 (FlowJo, OR, USA). A total of 100,000 events were recorded for the analysis. All experiments were replicated at least three times.

### Immunofluorescence Staining

HCEC cultured on chamber slides (Lab-Tek II Chamber Slide System; Nalge Nunc International) and treated with 2 nM rapamycin or vehicle control [dimethyl sulfoxide] (DMSO maximum concentration 0.04%) for 2 weeks. Then fixed in 4% paraformaldehyde (PFA) for 10 minutes and permeabilized with TBS + 0.3% Triton X-100 for 10 minutes at room temperature. The Cells washed three times with TBS + 0.03% Triton X-100 and incubated with 10% normal donkey serum in TBS containing 1% BSA for one hour at room temperature. Next, cells were incubated overnight at 4 °C with the rabbit anti-Ki67 (Abcam, Cambridge, MA), goat anti-human cytokeratin12 (sc-17101)(Santa Cruz Biotechnology, Santa Cruz, CA), ABCG2 rabbit mAb (42078) (Cell Signaling, Danvers, MA). Later cells washed with TBS + 0.03% Triton X-100 three times and incubated with secondary antibodies for 1 hour at room temperature. Cells were washed with TBS + 0.03% Triton X-100 and slides were mounted with an antifade reagent that contains 4′, 6-diamidino-2-phenylindole nuclear stain (ProLong Gold; Life Technologies) and permitted to dry overnight. The slides were visualized and photographed using immunofluorescence microscope (Carl Zeiss, Thornwood, NY), and photographed with an AxioCam (Carl Zeiss) camera. Cell staining intensities were measured using Image-J software (version 1.47, NIH) for individual cells and the distribution curve was drawn according to the intensity and cell number.

### Analysis of cell cycle by Flow cytometry

Starting at 10–15% confluency, HCEC were treated with rapamycin or vehicle control (DMSO) and continued until the cells reached 60–80% confluency. The spent media was removed and washed once with 1x phosphate buffered saline (PBS). The cells were collected using Trypsin/EDTA solution (Sigma-Aldrich) and centrifuged at 1500 rpm for five minutes at room temperature. The cell pellet was collected and gently resuspended in 1x PBS and fixed overnight at 4 °C in ice cold 70% ethanol. The next day they were centrifuged at 4000 rpm for five minutes at 4 °C discarding the supernatant. The cell pellet was washed twice with 1x PBS. Later, cells were incubated for 30 minutes at room temperature in 500 μl solution containing 0.05 mg/ml of propidium iodide (Life Technologies) and 0.05 mg/ml RNaseA (Thermo Scientific) in PBS and 0.1 m EDTA. Cells were analyzed by flow cytometry (BD LSR Fortessa) and cell cycle profiles were determined using Modfit software. All experiments were replicated at least three times.

### Cell Proliferation and Proliferative Potential

To measure cell proliferation, primary HCEC were plated onto 10 cm culture dishes (Corning Incorporated, Corning, NY) then grown to 10% to 15% confluence at which time rapamycin or DMSO treatment was started. Each day, starting with day one, three plates per condition were trypsinized and the total number of cells in each plate was counted. This was done for the next three consecutive days.

To measure proliferative potential, primary HCEC were treated with rapamycin or DMSO for one week after which the cells were trypsinized and plated onto 10 cm culture plates at a density of 5,000 cells/plate. The newly plated cells were only treated with keratinocyte serum-free medium (no rapamycin or DMSO) and followed for 10 days. The plates were then fixed and stained with crystal violet (Sigma-Aldrich). Colonies imaged with bright field microscopy (Leica DMi1) using LAS V4.5 software. The total intensity of the colonies in each plate was quantified using Image-J software (version 1.47, NIH) and the averages for each group were compared in Excel 2013 (Microsoft Corp).

### Real-Time PCR

The differential expression of selected genes was evaluated by quantitative PCR (qPCR) methods. Total RNA was extracted from cell culture using extraction reagent (TRIzol; Life Technologies) according to manufacturer’s protocol. After spectrophotometric assessment for quality and concentration (Nanodrop ND-1000; Thermo Scientific) the cDNAs were generated by using High Capacity cDNA Reverse Transcription Kit (Applied Biosystems, USA) using the manufacturer’s protocol. For each reaction, 2 μg of total RNA were used. The qPCR reactions were carried out in triplicate with FastStart Universal SYBER GREEN Master (Roche, Germany) in a total volume of 20 μL using thermal cycling condition of 10 minutes at 90 °C, 10 seconds at 95 °C followed by 40 cycles of 95 °C for 15 seconds and 60 °C for one minute. The PCR primers (all intron spanning) were 5′-AGCCAACCTTAACTGAGGAGT-3′ (Forward) and 5′-GGCAAGTTGATTGGAGGGATG-‘3 (Reverse) for N-Cadherin, 5′-GACCATTGAGGACCTGAGGA-3′ (Forward) and 5′-ATTGATGTCGGCTTCCACAC-3′ (Reverse) for Keratin-14, 5′-CTGGAAAACAATGCCCAGAC’-3 (Forward) and 5-‘GGGTGATGGAGAGAGAGCAT’-3 (Reverse) for DeltaNp63, 5-‘CAGGTCTGTTGGTCAATCTC’-3 (Forward) and 5-‘TCCTGTTGCATTGAGTCCTG’-3 (Reverse) for ABCG2 and 5-‘CTGCTCTGGGATTCTCTTCAG”-3 (Forward) and 5-“ATCTTCCTCAGCTTGTCCATG”-3 (Reverse) for IL-1β and 5-“GTGTAAACATGACTTCCAAGCTG”-3 (Forward) and 5-“AAACTTCTCCACAACCCTCTG”-3 (Reverse) for IL-8 and 5-“CTTCTCCTTCCTGATCGTGG”-3 (Forward) and 5-“GCTGGTTATCTCTCAGCTCCA”-3 (Reverse) for TNF-α. Ribosomal RNA 18 s was used as an endogenous control. All qPCR reactions were replicated at least three times.

### Inflammatory gene induction

HCEC were treated with rapamycin or DMSO for one week then exposed to the TLR3 agonist polyinosinic-polycytidylic acid (poly-IC) (Invitrogen, San Diego, California) 25 μg/ml for three days. Cells were washed with PBS, the RNA extracted and qPCR was done for TNFα, IL-1β, and IL-8 as described above.

### Senescence-Associated Beta-Galactosidase (X-β-Gal SA) Staining

X-gal staining for β-galactosidase activity was performed on HCEC treated with rapamycin 2 nM and control (DMSO) at week two and week five. Each sample was rinsed twice with PBS, fixed in 3.7% PFA for 15 minutes. Cells were washed with PBS, and then stained for 36 hours at 37 °C in X-gal solution with PH of 6.0 containing 1 mg/ml X-gal (Thermo Scientific), 5 mM potassium ferricyanide, 5 mM potassium ferrocyanide, 2 mM MgCl_2_. X-gal positive cells (cells with greenish color) were examined in 10 fields from each sample under light microscope (Carl Zeiss, Thornwood, NY), and photographed with an AxioCam (Carl Zeiss) camera.

### TUNEL assay

TUNEL (Terminal deoxynucleotidyl transferase dUTP nick end labeling) assay was performed to detect apoptotic cells in HCEC treated with rapamycin or control, after two weeks using ApopTagPlusFluoresceinInSituKit (Millipore, Billerica, MA, USA). Briefly, the media was removed and cells were fixed in 1% PFAinPBS (pH 7.4) for 10 minutesatroom temperature. Permeabilized cells in were cooled in ethanol after washing them with PBS, equilibration buffer applied and then TdT enzyme added and incubated at 37 °C for one hour. The cells were agitated for 15 second in wash buffer then washed with PBS. Digoxigenin-fluorescein conjugate was added and incubated at room temperature for 30 minutes in the dark. The cells were washed with PBS, mounted with DAPI and visualized with immunofluorescence microscope (Carl Zeiss, Thornwood, NY), and photographed with an AxioCam (Carl Zeiss) camera.

### Statistical Analyses

For descriptive statistical analysis we used Student’s t-test. Results are reported as the mean ± standard deviation (SD). Statistical significance was set at P ≥ 0.05.

## Additional Information

**How to cite this article**: Gidfar, S. *et al*. Rapamycin Prolongs the Survival of Corneal Epithelial Cells in Culture. *Sci. Rep.* **7**, 40308; doi: 10.1038/srep40308 (2017).

**Publisher's note:** Springer Nature remains neutral with regard to jurisdictional claims in published maps and institutional affiliations.
